# AI-enhanced virtual screening identifies a potent small-molecule modulator of ClC-3 for cervical cancer drug discovery

**DOI:** 10.3389/fbinf.2026.1887419

**Published:** 2026-08-07

**Authors:** Chao Liu, Chongxing Ji

**Affiliations:** 1 School of Artificial Intelligence, Dongguan City University, Dongguan, Guangdong, China; 2 Faculty of Applied Sciences, Macao Polytechnic University, Macau, Macao SAR, China

**Keywords:** CLC-3 chloride channel, deep-learning rescoring, lead compound discovery, molecular dynamics simulation, virtual screening

## Abstract

ClC-3 chloride channels play essential roles in cervical cancer progression by regulating lysosomal acidification, cell volume homeostasis, and chemoresistance. However, no highly selective small-molecule modulators of ClC-3 have been reported to date. Motivated by the urgent clinical need to reverse ClC-3-mediated chemoresistance and the challenge of processing massive chemical libraries under limited computational hardware resources, we propose a novel AI-driven Drug Discovery (AIDD) pipeline. Here, we present an integrated virtual drug discovery framework that transitions from traditional Computer-Aided Drug Design (CADD) by combining large-scale molecular docking, deep-learning-based rescoring, pharmacokinetic filtering, and atomistic molecular dynamics (MD) simulations. The primary advantage of this proposed scheme lies in the integration of GNINA 3D-convolutional neural network (CNN) rescoring, which significantly reduces the false-positive rates inherent in empirical scoring functions for membrane proteins. A library of ∼180,000 ZINC15 compounds was initially screened using AutoDock Vina, followed by GNINA convolutional neural network rescoring to refine predicted binding affinity and pose confidence. ADMET profiling further narrowed the candidates, providing computational proof of drug-likeness and toxicity criteria rather than experimental validation. Ultimately, only ZINC000001556308 (Lig8) satisfied all *in silico* criteria. To validate binding stability, we performed 100-ns all-atom MD simulations of the ClC-3–Lig8 complex embedded in a lipid bilayer. Lig8 induced reduced RMSD fluctuations, lower RMSF values across key transmembrane helices, and a more compact radius of gyration, indicating enhanced structural stabilization of ClC-3. MM/PBSA calculations confirmed favorable binding energetics dominated by van der Waals interactions, while per-residue decomposition identified PHE527, GLY283, and GLY584 as major contributors to ligand recognition. These results reveal a previously uncharacterized binding pocket within the ClC-3 transmembrane domain and highlight Lig8 as a promising lead compound for targeting ClC-3-mediated oncogenic signaling. Overall, this study establishes the first comprehensive computational framework for ClC-3 modulator discovery and provides a validated chemical scaffold for future therapeutic development against cervical cancer.

## Introduction

Cervical cancer (CVC) (Sean, 2025) is a leading gynecological malignancy characterized by uncontrolled cellular proliferation, invasive metastasis, and chemoresistance. Despite advances in HPV vaccination and early screening (Arbyn, 2025), the 5-year survival rate for metastatic CVC remains below 20%, underscoring an urgent need for targeted therapeutics (Lin et al., 2025). Recent studies highlight ClC-3 (Chloride Channel 3) chloride ion channels as pivotal regulators of CVC pathogenesis through mechanisms including lysosomal acidification modulation (Chen et al., 2025), PI3K/Akt/mTOR pathway activation (Jiayi, 2025), and tumor stemness maintenance.

**FIGURE 1:**
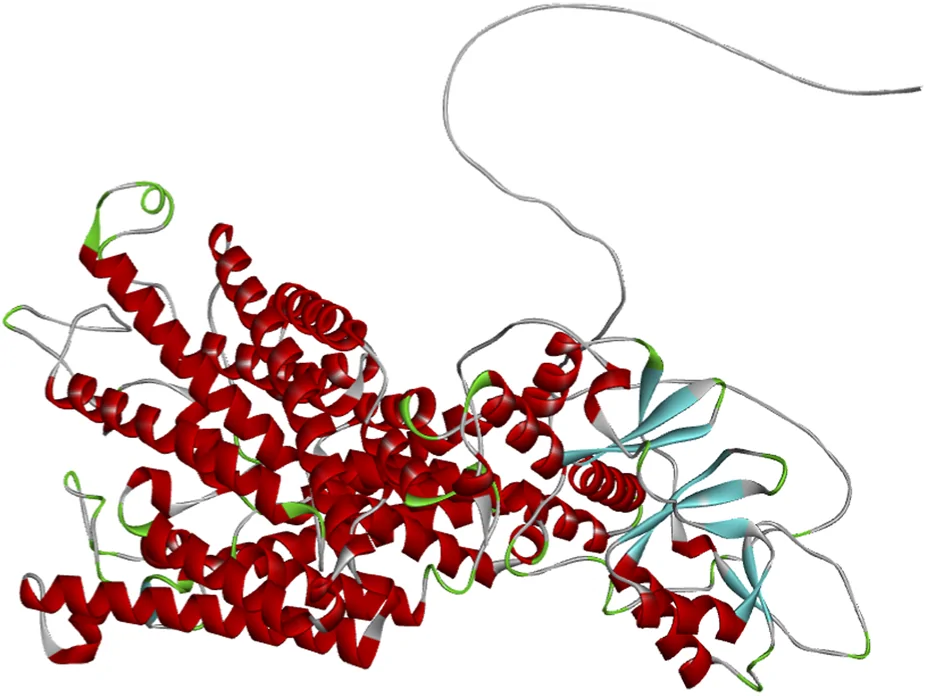
Schematic representation of the ClC-3 chloride ion channel protein. The ClC-3 chloride ion channel protein structure is acquired from Unirpot database predicted by AlphaFold.

ClC-3, a voltage-gated anion channel encoded by the *CLCN3* gene, is abundantly expressed in CVC tissues and correlated with advanced tumor stage, lymphovascular invasion, and poor prognosis (Zhang et al., 2024; Liu et al., 2024; Zhang et al., 2023). Structural studies reveal its functional domains include transmembrane helices for ion permeation and a cytoplasmic C-terminus regulating channel gating (Figure 1) (Schrecker et al., 2025; Di Fruscio et al., 2024). Dysregulated ClC-3 activity promotes cisplatin resistance via V-ATPase-mediated lysosomal alkalinization and autophagy suppression (Shen et al., 2025; Cotter et al., 2022), making it a promising therapeutic target. However, the complexity of CVC molecular pathogenesis and limited validated targets have hindered drug discovery (Xia et al., 2025; Ruxandra, 2024; Victoria Sánchez-Meza et al., 2025). Despite the growing recognition of ClC-3 as a key regulator of cervical cancer progression, current pharmacological tools like IAA-94 lack target specificity. Consequently, no validated small-molecule drug modulator of ClC-3 is currently available, and the structural basis of ligand recognition remains poorly defined. This knowledge gap significantly limits the development of targeted therapeutics.

Virtual drug screening (VDS) offers a transformative approach for ion channel-targeted drug development. By integrating molecular docking, pharmacokinetic modeling, and molecular dynamics (MD) simulations, VDS enables high-throughput identification of candidate compounds with optimal binding affinity and pharmacokinetic profiles (Nguyen et al., 2024; Jamrozik et al., 2024; Zare et al., 2024). This method has demonstrated success in cancer research—for example, discovering EGFR inhibitors for non-small cell lung cancer and PARP inhibitors for BRCA-mutant ovarian cancer (Wang, 2024; Kranthi Reddy et al., 2025; Al Kury, 2025). Nevertheless, its application to CVC remains underexplored due to target validation challenges and tumor heterogeneity (Zhao, 2025; Singh and Patel, 2024).

**FIGURE 2:**
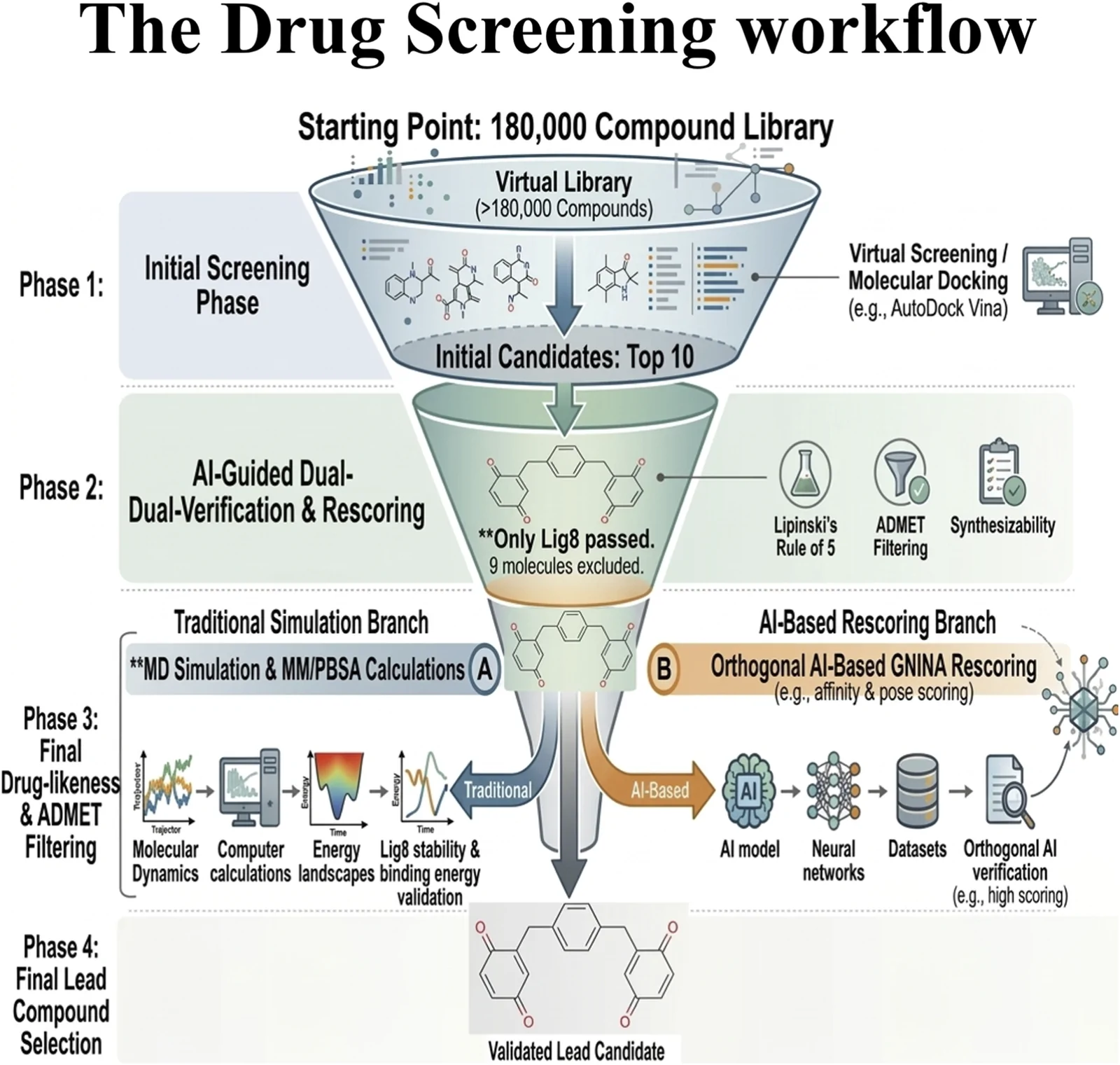
Schematic representation of the hierarchical virtual screening and multi-faceted validation workflow for lead compound discovery. The protocol encompasses four primary phases: (Phase 1) Initial Screening: High-throughput molecular docking (e.g., AutoDock Vina) of a virtual library containing >180,000 compounds to identify the top 10 preliminary candidates. (Phase 2) AI-Guided Verification & Rescoring: Rigorous filtering of the initial candidates based on Lipinski’s Rule of Five, ADMET properties, and synthesizability, which isolated Lig8 as the sole surviving molecule. (Phase 3) Dual-Branch Validation: Comprehensive evaluation of Lig8 utilizing two parallel methodologies: **(A)** a traditional computational branch employing Molecular Dynamics (MD) simulations and MM/PBSA calculations to assess structural stability and binding energy landscapes; and **(B)** an orthogonal AI-based branch utilizing deep learning models (e.g., GNINA) for precise affinity and pose rescoring. (Phase 4) Final Selection: Convergence of the validation branches confirms the final validated lead candidate.

The overarching motivation of this study is to discover highly selective small-molecule modulators that can target ClC-3 and potentially reverse chemoresistance in cervical cancer. Traditional virtual drug screening (VDS) often struggles with high false-positive rates when applied to complex membrane proteins and demands extensive hardware resources for large-scale library screening. To address these challenges, we pioneered a multi-stage AI-driven virtual drug discovery workflow (Figure 2). Unlike conventional protocols, our proposed scheme integrates deep-learning-based GNINA 3D-CNN rescoring. The primary advantage of this novel technique is its ability to extract spatial and chemical features of protein-ligand complexes directly from 3D grids, providing a more robust evaluation of protein-ligand interactions and effectively bypassing the hardware limitations of traditional exhaustive CADD approaches. However, we also acknowledge the inherent disadvantages of this AI-based scheme, such as its reliance on the quality of training datasets and its relatively “black-box” nature compared to physically intuitive empirical scoring functions. To mitigate these limitations and validate the dynamic stability of the AI-predicted poses, we incorporated atomistic MD simulations and MM/PBSA calculations (Khalid et al., 2025; Abdel-Aziz, 2024; Smith and Jones, 2024) after AMDET prediction (Baig et al., 2025; Xiong et al., 2021; Wang et al., 2025).

In summary, this study represents the first systematic computational exploration of ClC-3 ligandability. By identifying a previously uncharacterized binding pocket within the transmembrane domain and proposing a validated chemical scaffold, this work bridges computational innovation with translational oncology, offering a promising pathway and lead compounds for precision therapeutics against cervical cancer.

## Materials and methods

### Dataset preparation

Approximately 180,000 complex molecules, each featuring a unique 3D framework, were sourced from the “In-Stock” and “Standard” subsets of the extensive ZINC15 database (https://zinc15.docking.org/). The selection criteria were rigorously restricted to specific tranches to ensure lead-like properties: molecular weight (MW) between 300 and 350 Da, calculated LogP between −1.0 and 3.5, and a net charge of 0 (neutral) at reference physiological pH. Each molecule served as a standard, readily accessible, and neutral drug candidate. Following meticulous evaluation, the protonation states of these ligands were determined, and key hydrogen atoms along with partial charges were added to ensure their suitability for subsequent analyses.

### Molecular docking

The 3D coordinates of the ClC-3 chloride ion channel protein structure were obtained from the AlphaFold Protein Structure Database (Uniprot ID: P51790). Prior to molecular docking, the protonation state of the protein was determined and polar hydrogen atoms were explicitly added to match the force field requirements of Autodock Vina. Non-polar hydrogen atoms were merged into adjacent heavy atoms according to the Autodock4 algorithm specifications to ensure compatibility with the docking protocol.

Small-molecule ligands were prepared using Open Babel to generate conformers and assign Gasteiger-Marsili partial charges, a method validated to reproduce about 80% of co-crystallized ligand poses in benchmark studies. The ligands were docked into the CIC-3 transmembrane domain using Autodock Vina v1.2.0. The docking grid box was centered on the proposed chloride binding pocket with coordinates X = 111.949, Y = 85.764, and Z = 96.195. The box dimensions were set to 47.25 × 47.25 × 47.25 Å^3^ to fully encompass the binding interface. The global search exhaustiveness parameter was set to the default value of 8.

Given the extensive scale of the virtual screening library, a single docking run was performed for each compound to maximize computational efficiency. During this run, the algorithm was configured to output 10 binding modes (num_modes = 10), and the top-ranked pose with the lowest binding affinity was selected for deep learning rescoring. To enhance computational efficiency for large-scale virtual screening, we implemented the parallelized MPI-Vina wrapper (freely available at https://github.com/mokarrom/mpi-vina) to achieve a multifold acceleration compared to single-core CPU execution.

### Deep learning-based rescoring

To mitigate the inherent false-positive rates of traditional scoring functions and further validate the binding poses, the top screened candidates were rescored using GNINA1.3.2, a deep learning framework based on 3D convolutional neural networks (3D-CNNs). The rescoring was performed using the pre-trained crossdock2020 ensemble model. To balance computational efficiency and scoring accuracy, the command-line argument--cnn_scoring rescore was explicitly specified, applying the 3D-CNN models exclusively to evaluate and re-rank the final poses generated by the empirical scoring function. For rigorous validation, IAA-94 (a known ClC-3 blocker) was utilized as a positive control, due to its widespread historical use as a reference blocker for ClC channels, despite its known lack of strict isoform specificity. Glucose was selected as a negative control because it is a biologically inert, highly polar molecule with no anticipated binding affinity to the hydrophobic transmembrane domains of ion channels. Both the CNN_Affinity (predicting binding affinity in pKd/pKi) and CNN_Pose_Score (evaluating the probability of a native-like binding conformation) were extracted to assess the candidates.

## ADMET prediction

ADMET properties are pivotal in assessing the drug-like potential of compounds, as they directly influence pharmacokinetics, safety profiles, and ultimate therapeutic efficacy. For the ClC-3 chloride channel protein virtual screening campaign, we integrated state-of-the-art computational ADMET prediction frameworks to rigorously evaluate candidates from the docking-derived hits. Specifically, the SwissADME (http://www.swissadme.ch) platform was employed to assess key ADME parameters using ensembles of machine learning models trained on diverse chemical libraries. Complementary toxicity profiling was conducted via ADMETLab (https://admetmesh.scbdd.com), which leverages deep learning architectures validated against experimental toxicity datasets to predict chronic toxicity, mutagenicity, and cardiotoxicity endpoints. This dual-tool approach ensures comprehensive coverage of both pharmacokinetic liabilities and safety hazards, enabling the systematic prioritization of compounds that not only exhibit strong binding affinity to ClC-3 but also possess favorable drug-like properties and minimal toxicity risks. The integration of these *in silico* ADMET predictors aligns with modern drug discovery paradigms, where early-stage bioavailability and toxicity assessment significantly enhances the efficiency of lead optimization and reduces attrition rates in later development stages.

### Energy minimization

Energy minimization was utilized to refine the structural conformations of candidate compounds and ClC-3 chloride ion channel models. This procedure is essential for ensuring precise molecular docking simulations and accurate binding energy calculations. Following initial structural preparation, both ligands and receptor models underwent energy minimization protocols implemented via Gromacs. The process systematically adjusts atomic coordinates and bond geometries to attain a configuration of minimal potential energy, representing a thermodynamically stable state.

During minimization, constraints were strategically applied to maintain critical molecular features, including the preservation of covalent bond integrity and the geometric accuracy of functional groups. The optimization employed gradient-based algorithms—such as steepest descent, conjugate gradients, and L-BFGS—to compute atomic forces and iteratively reposition atoms to minimize the total potential energy. These methods ensure convergence toward a local energy minimum while maintaining chemical realism.

By executing energy minimization, we derived optimized conformations for both drug candidates and ClC-3 receptor models. These refined structures were subsequently employed in molecular dynamics (MD) simulations, enhancing the precision and robustness of the virtual screening pipeline. This approach facilitated the identification of high-affinity, selective ligands targeting the ClC-3 chloride ion channel, advancing the discovery of potential therapeutic agents for chloride channel-associated disorders.

### Equilibration

Following energy minimization, the optimized ClC-3 chloride ion channel receptor models and candidate ligands underwent equilibration procedures to establish thermodynamic equilibrium prior to molecular dynamics (MD) simulations. This step is critical for stabilizing the systems in a solvent-optimized environment, ensuring accurate representation of physiological conditions.

Equilibration was performed using Gromacs with periodic boundary conditions and validated force fields tailored to membrane proteins and ion channels. The simulations incorporated explicit solvent models (e.g., TIP3P water) and physiological ionic strength (150 mM NaCl) to mimic native cellular environments. System temperatures were maintained at 298.15 K via velocity-rescale thermostats, while pressure was controlled at 1 atm using Parrinello-Rahman barostats to simulate *in vivo* conditions.

The equilibration protocol comprised two sequential phases:

1. NVT equilibration (constant particle number, volume, and temperature): A 100-ps simulation was first conducted to thermalize the system, allowing atomic velocities to equilibrate while constraining heavy-atom positions using harmonic restraints on the protein backbone and ligand core atoms. This phase ensured gradual adaptation to the target temperature without disruptive conformational changes.
2. NPT equilibration (constant particle number, pressure, and temperature): Following NVT, a 100-ps NPT simulation was executed to achieve pressure equilibrium. The system volume was allowed to adjust isotropically, enabling the solvent and membrane components to pack efficiently around the ClC-3-ligand complexes. Restraints were gradually reduced to permit controlled relaxation of side chains and solvent orientation.

Through these equilibration steps, stable conformations of the receptor-ligand complexes were obtained, characterized by converged Root Mean Square Deviation (RMSD) and stable energetic profiles.

### MD simulation

The molecular dynamics (MD) simulation framework was constructed using initial coordinates derived from AlphaFold-predicted structures of ClC-3 chloride channel (Uniprot ID: 51790) accessed via the Uniprot database. System preparation and equilibration were executed with GROMACS 2019.6 software, employing the AMBER99SB-ILDN force field for protein parametrization and TIP3P water model for solvent hydration. The integration time step was set to 2 fs, with all bonds involving hydrogen atoms constrained using the LINCS algorithm. Long-range electrostatic interactions were treated using the Particle Mesh Ewald (PME) method. Short-range van der Waals and electrostatic interactions were both calculated with a cut-off radius of 1.2 nm, utilizing a force-switch modifier starting at 1.0 nm for van der Waals forces. The CHARMM-GUI web server was utilized to embed the protein within a DPPC lipid bilayer membrane, with sodium counterions added to neutralize the system charge. Visualization and trajectory analysis were performed using VMD and XMGRACE packages.

RMSD was calculated to quantify global structural stability throughout the simulation. The metric measures the average displacement of Cα atoms relative to the AlphaFold-predicted reference structure:

$$RMSD=\sqrt{\frac{\sum_{i=0}^{N}\left[m_{i}{*\left(X_{i}-Y_{i}\right)}^{2}\right]}{M}}$$

where *N* denotes the total number of atom pairs, *i* denotes the *ith* atom pair, *M* denotes the total weight sum, *m*_*i*_ denotes the weighting factor (the relative importance of specific *ith* atomic pairs), *X*_*i*_ and *Y*_*i*_ denote the reference coordinate and target coordinate of the *i*th atom, respectively.

Root Mean Square Fluctuation (RMSF) was employed to characterize regional flexibility:

$$RMSF=\sqrt{\frac{1}{T}\sum_{t=1}^{T}\sum_{i=1}^{N}{\left[r_{i}\left(t\right)-\langle r_{i}\rangle \right]}^{2}}$$

where *T* denotes the total simulation time steps, *r*_*i*_(*t*) denotes the instantaneous position of atom *i* at time *t* and $\langle r_{i}\rangle$ denotes the time-averaged position of atom *i*. Elevated RMSF values in loop regions (e.g., extracellular domains) versus conserved α-helical bundles provide insights into allosteric communication pathways and ligand-binding hotspots.

The moment of inertia, alternatively termed rotational inertia or angular mass, quantifies an object’s resistance to rotational acceleration about a specified axis. It mathematically encodes the spatial distribution of mass relative to an axis of rotation, where greater values correspond to enhanced resistance against changes in rotational velocity. In rotational dynamics, this property fundamentally governs relationships involving torque, angular momentum conservation, and rotational kinetic energy. The radius of gyration (symbolized as *k*), which provides equivalent rotational behavior through a concentrated mass model, serves as its geometric representation:

$$k=\sqrt{\frac{I}{m}}$$

where *I* represents the moment of inertia and *m* represents mass respectively.

This integrated MD approach enables atomic-level characterization of ClC-3 dynamics, facilitating the identification of ligand-binding modes that balance high affinity with minimal conformational perturbation—critical criteria for virtual screening hit prioritization in chloride channel modulator discovery.

### Per-residue energy decomposition using MM/PBSA

Per-residue energy decomposition via MM/PBSA provides atomic-level insights into ligand binding mechanisms by quantifying the energetic contribution of each protein residue to the total binding free energy. For the ClC-3 chloride ion channel protein, this method was applied to identify critical residues governing ligand recognition in the transmembrane domain.

The total binding free energy (*ΔG*_*binding*_) was decomposed as:

$$\Delta G_{binding}=\Delta E_{MM}+\Delta G_{PB}+\Delta G_{non-polar}-T\Delta S$$

where *ΔE*_*MM*_ represents the molecular mechanics energy (sum of internal, van der Waals, and Coulombic terms), *ΔG*_*PB*_ denotes the Poisson-Boltzmann energy, and ΔG_non-polar_ is the non-polar solvation energy, which is in linear relationship with the solvent-accessible surface area (SASA):

$$\Delta G_{non-polar}=\gamma SASA+\beta$$

The entropic contribution (-*TΔS*) was omitted in this study due to the substantial computational cost and potential inaccuracies introduced by normal mode analysis for large membrane protein systems.

## Results

### Virtual screening

Within the extensive ZINC15 database, we explored ∼180,000 potential drug candidates. Through advanced virtual screening methodologies, we identified elite molecules targeting the receptor. From this pool, 10 top-tier compounds emerged with binding affinities ranging from −9.7 to −10.2 kcal/mol (Table 1). We then conducted a comprehensive pharmacokinetic (ADME) and toxicity assessment of these candidates to optimize therapeutic selection.

**TABLE 1:** Docking results of MPI-Vina between protein and ligands.

ZINC ID	Short name in this study	Binding affinity (kcal/mol)
ZINC000013923892	Lig1	−10.2
ZINC000252641347	Lig2	−9.9
ZINC000004405600	Lig3	−9.8
ZINC000013577161	Lig4	−9.8
ZINC000004384802	Lig5	−9.8
ZINC000186886036	Lig6	−9.8
ZINC000004697447	Lig7	−9.8
ZINC000001556308	Lig8	−9.7
ZINC000254496813	Lig9	−9.7
ZINC000013759087	Lig10	−9.7

### GNINA rescoring validates Lig8 as a potent ClC-3 modulator

Following the initial virtual screening, we employed GNINA deep learning rescoring to rigorously evaluate the top 10 candidates. As detailed in Table 2, while the traditional empirical affinity score struggled to distinguish the negative control (−6.81 kcal/mol) from the positive control (−5.86 kcal/mol), the deep learning-based CNN affinity score successfully discriminated IAA-94 (5.39) from glucose (3.66), strongly validating the reliability and necessity of our docking protocol. Building on this validated model, the top 10 hit compounds (Lig1-Lig10) exhibited robust binding profiles. Notably, molecules such as Lig10 (affinity = −8.21 kcal/mol) and Lig9 (CNN affinity = 5.51) demonstrated superior or comparable docking performance to the established inhibitor IAA-94 across multiple scoring metrics. These highly favorable CNN scores and binding affinities suggest that the identified top 10 ligands possess significant potential as potent ClC-3 channel blockers for cervical cancer therapy.

**TABLE 2:** Molecular docking results of the top 10 hit compounds, alongside positive (IAA-94) and negative (Glucose) controls, against the ClC-3 chloride ion channel using GNINA. The table presents the empirical Vina affinity (kcal/mol), CNN pose score (probability of the pose being active-like), and CNN affinity (predicted binding affinity).

Ligand name	Affinity (kcal/mol)	CNN pose score	CNN affinity
Glucose	−6.81	0.69	3.66
IAA-94	−5.86	0.79	5.39
Lig1	−7.39	0.44	5.04
Lig2	−7.62	0.63	4.92
Lig3	−6.21	0.77	4.78
Lig4	−6.47	0.76	4.75
Lig5	−7.46	0.51	5.20
Lig6	−7.92	0.82	5.39
Lig7	−7.28	0.60	4.88
Lig8	−7.51	0.77	4.83
Lig9	−7.87	0.85	5.51
Lig10	−8.21	0.73	5.23

**FIGURE 3:**
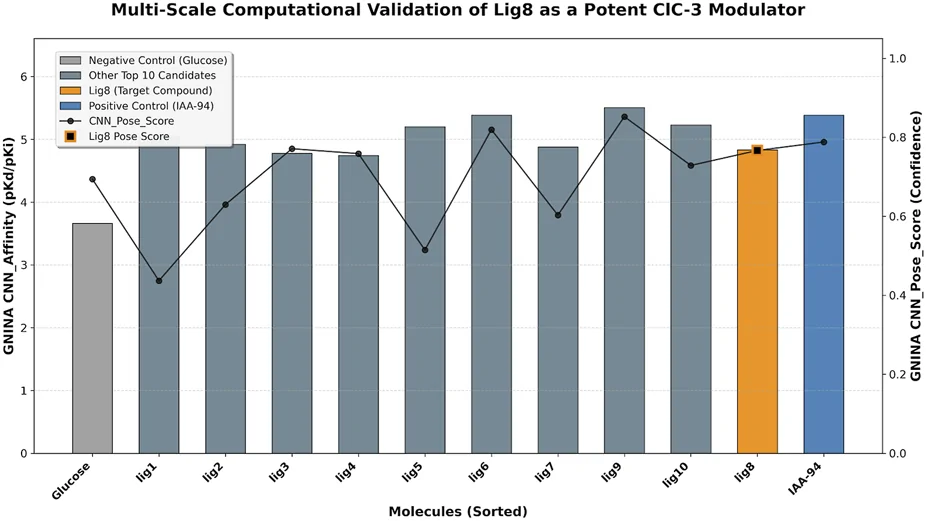
Deep learning-based rescoring of the top candidate compounds using GNINA. The bar chart (left Y-axis) represents the predicted CNN_Affinity (pKd/pKi), indicating the binding strength. The line graph (right Y-axis) displays the CNN_Pose_Score, representing the neural network’s confidence in the docked pose. Glucose and the known ClC-3 inhibitor IAA-94 were included as negative and positive controls, respectively. The primary target compound, Lig8, is highlighted in orange.

As illustrated in Figure 3, the rescoring model demonstrated excellent discriminative capability: the negative control (glucose) exhibited the lowest CNN_Affinity and a mediocre pose score, whereas the positive control (IAA-94) showed high binding affinity and strong pose confidence. Notably, our target compound, Lig8, emerged as a promising computational modulator. It achieved a CNN_Affinity of 4.83 pK d/pKi and a high CNN_Pose_Score of 0.77. These metrics are highly comparable to the established inhibitor IAA-94, indicating a strong and stable binding potential to the ClC-3 channel.

**FIGURE 4:**
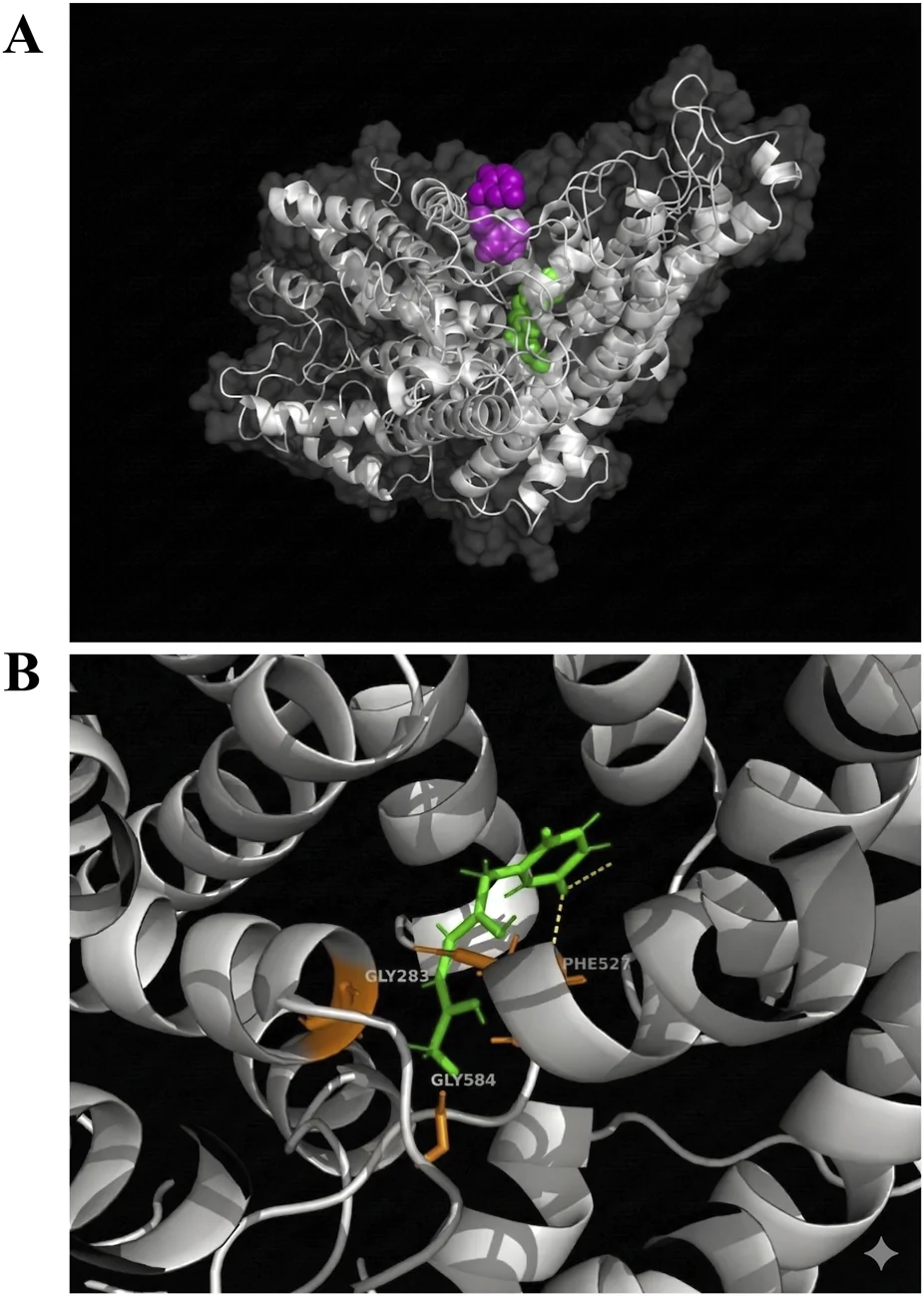
Comparison of the molecular binding modes before and after molecular dynamics (MD) simulation. **(A)** Structural superimposition of Lig8 bound to the ClC-3 protein. The purple spheres represent the initial binding pose predicted by GNINA docking, while the green spheres represent the stabilized conformation obtained after the MD simulation. **(B)** Detailed local view of the stabilized non-covalent interactions between the post-MD ligand (green sticks) and the surrounding amino acid residues of ClC-3. Key interacting residues, including GLY283, PHE527, and GLY584, are highlighted as orange sticks. Yellow dashed lines indicate critical hydrogen bonds and π-π stacking interactions.

To deeply elucidate the inhibitory mechanism of the identified hit compound against the ClC-3 chloride ion channel protein, detailed molecular docking analysis was conducted, as illustrated in Figure 4. The overall structural perspective (Figure 4A, top) confirms that the compound effectively anchors into the active binding pocket of the ClC-3 protein. A closer examination of the interaction interface (Figure 4B, bottom) reveals that the stability of the protein-ligand complex is predominantly maintained by a network of key interactions with surrounding amino acid residues. Specifically, the ligand is positioned in close proximity to GLY283, PHE527, and GLY584. The compound forms critical intermolecular interactions (indicated by yellow dashed lines) with these residues, such as potential π-π stacking or hydrophobic contacts with the aromatic ring of PHE527, and hydrogen bonding with GLY283 and GLY584. These specific interactions are pivotal for firmly securing the ligand within the ClC-3 channel, which may consequently restrict the conformational transitions necessary for chloride ion transport, thereby exerting its potential anti-cervical cancer efficacy.

### ADMET prediction

In this study, we leveraged the SwissADME server to thoroughly investigate the critical ADME properties of our compounds. Specifically, we examined their lipophilicity—defined as their capacity to dissolve in fats, oils, and nonpolar solvents—along with their aqueous solubility and overall drug-likeness. These parameters, detailed in Table 3, provide essential insights into the potential druggability of our top 10 ligands (designated as Lig1 to Lig10 in Table 1). Specifically, drug-likeness was strictly evaluated against Lipinski’s Rule of Five thresholds (MW ≤ 500 Da, LogP ≤5, H-bond donors ≤5, and H-bond acceptors ≤10). Additionally, synthetic accessibility (SA) was evaluated on a scale from 1 (very easy) to 10 (very difficult), with all candidates scoring well below 5, indicating high synthesizability. Our results demonstrate that all these compounds display encouraging characteristics for drug development. Importantly, it should be noted that these profiling platforms provide predicted computational proof of drug-likeness and toxicity criteria, rather than real experimental validation.

**TABLE 3:** List of ADME properties of top 10 ligands.

Properties		Lig1	Lig2	Lig3	Lig4	Lig5	Lig6	Lig7	Lig8	Lig9	Lig10
Physicochemical properties	MW(g/mol)	329.32	323.31	309.28	315.37	342.35	345.39	344.36	318.32	317.34	317.34
	Heavy atoms	25	24	23	24	26	26	26	24	24	24
	Arom. heavy atoms	19	17	17	12	18	12	12	6	15	15
	Rotatable bonds	2	5	5	2	1	3	2	4	2	2
	H-bond acceptors	5	6	6	2	3	3	4	4	3	3
	H-bond donors	2	1	1	0	3	2	0	0	2	2
Lipophilicity	Log Po/w	1.67	1.93	1.76	3.06	1.9	2.53	2.93	2.3	2.64	2.64
Water solubility	Log S (ESOL)	Soluble	Moderate	Soluble	Soluble	Soluble	Soluble	Soluble	Soluble	Moderate	Moderate
Pharmacokinetics	GI absorption	High	High	High	High	High	High	High	High	High	High
Drug-likeness	Lipinski	Yes	Yes	Yes	Yes	Yes	Yes	Yes	Yes	Yes	Yes
Medi. Chemistry	Synth. Accessibility	3	3.06	2.94	3.6	4.05	2.99	4.14	2.8	2.98	2.98

Nevertheless, modern drug discovery is a multifaceted process that requires the strict exclusion of undesirable safety liabilities to reduce late-stage attrition. To address this, we employed ADMETLab 2.0 to assess our top 10 compounds for drug-induced hERG channel blockade, AMES mutagenicity, and carcinogenic potential. For these specific endpoints, evaluations were based on the model’s output probabilities, utilizing a strict threshold where >0.5 indicates a high risk (“Yes”) and <0.5 indicates a low risk (“No”). Rather than employing a weighted scoring system that might offset toxicity with high binding affinity, we applied a strict hard-filter approach. Any compound failing a single toxicity metric was definitively eliminated, even if the failure was marginal. As presented in Table 4, a critical comparative analysis of the top candidates highlights the necessity of this safety profiling. While several candidates—particularly Lig6, Lig9, and Lig10—demonstrated highly favorable binding affinities and strong GNINA CNN scores, they were strictly excluded from further consideration due to critical toxicity flags. Specifically, Lig9 and Lig10 exhibited high risks for both AMES mutagenicity and carcinogenicity, and Lig6 presented a carcinogenic liability. In contrast, only ZINC000001556308 (Lig8) successfully passed all rigorous toxicity evaluations without exhibiting any hERG, AMES, or carcinogenic liabilities. Consequently, because Lig8 achieved the optimal balance of strong target engagement and a clean safety profile, it was prioritized as the sole promising computational lead for our subsequent molecular dynamics validation.

**TABLE 4:** List of the drug-induced hERG inhibition, AMES toxicity, carcinogens of top 10 ligands.

ZINC ID	hERG inhibition	AMES	Carcinogens
ZINC000013923892	No	No	Yes
ZINC000252641347	No	Yes	Yes
ZINC000004405600	No	Yes	Yes
ZINC000013577161	No	No	Yes
ZINC000004384802	Yes	Yes	Yes
ZINC000186886036	No	No	Yes
ZINC000004697447	No	Yes	No
ZINC000001556308	No	No	No
ZINC000254496813	No	Yes	Yes
ZINC000013759087	No	Yes	Yes

### Energy minimization and equilibration

**FIGURE 5:**
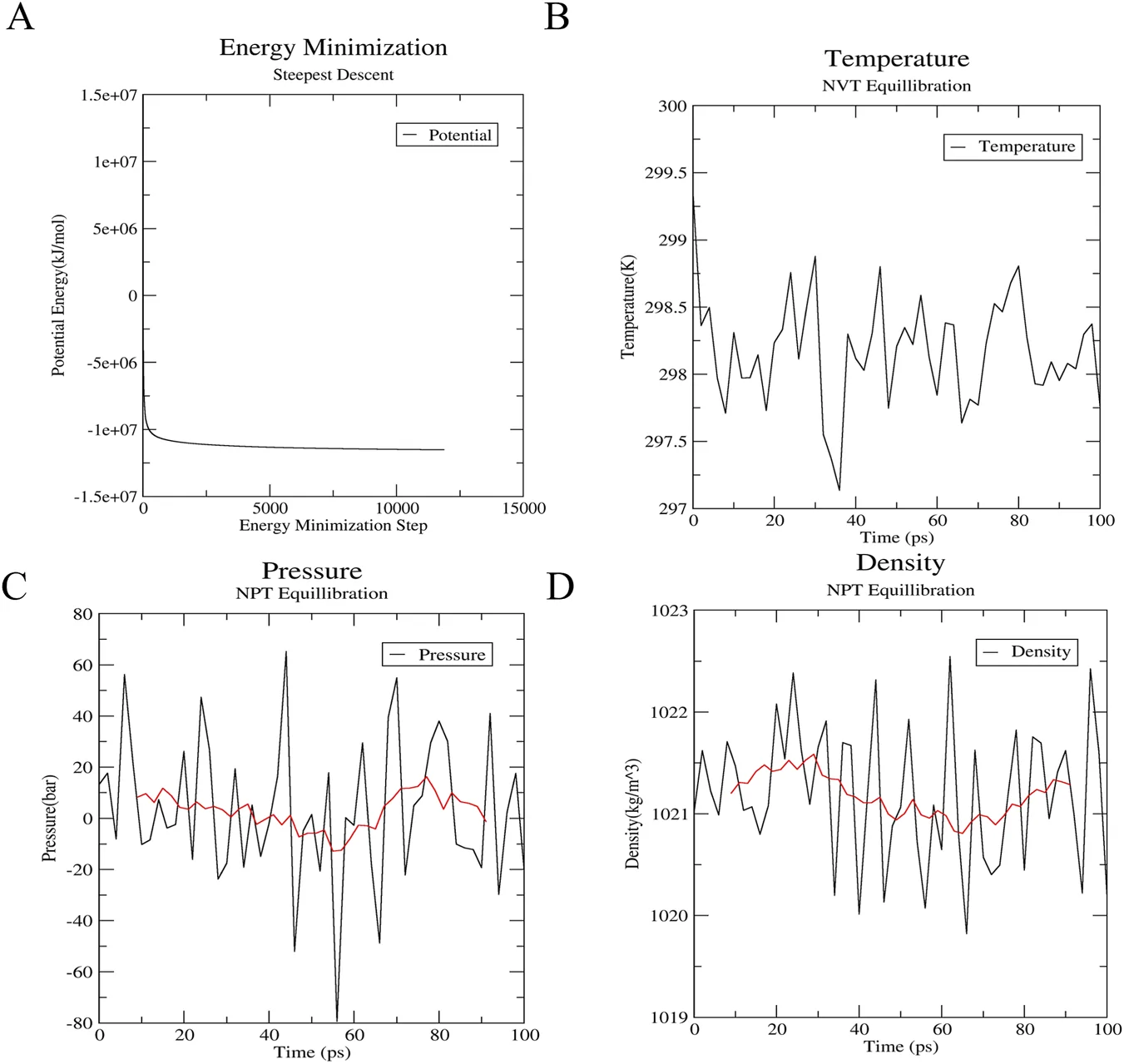
The energy minimization and equilibration step before MD simulation. **(A)** The variation of potential energy with energy minimization step. **(B)** The variation of temperature within 100 ps **(C)** The variation of pressure within 100 ps? **(D)** The variation of density within 100 ps.

The simulation framework was meticulously designed, employing the AMBER99SB-ILDN force field for proteins and the TIP3P water model. A cubic water box encapsulated and solvated the protein-water system, initiating multiple refinement stages. First, an energy minimization protocol of approximately 12,000 steps optimized system energetics using a steepest descent integrator with a fine 0.001 ps time step. As shown in Figure 5A, the potential energy rapidly declined from 1.50 × 10^7^ kJ/mol to −1.05 × 10^7^ kJ/mol within 100 steps, gradually approaching to a well-converged system with a minimized energy of −1.10 × 10^7^ kJ/mol.

Post-minimization, the system underwent controlled heating as seen in Figure 5B. Under NVT conditions, temperature was gradually elevated to 300K over 100 ps, with initial velocities sampled from a Maxwellian distribution. Temperature exhibited initial fluctuations before stabilizing near 300K, demonstrating thermal equilibrium.

For pressure equilibration in the NPT ensemble (Figure 5C), the Parrinello-Rahman barostat maintained pressure coupling, yielding an average pressure of 1.0 ± 40.1 bar (reference: 1 bar). The observed root-mean-square fluctuation is typical in MD simulations, rendering the minor deviation statistically insignificant. The 10-ps running average effectively tracks pressure evolution.

Simultaneously, system density was monitored (Figure 5D). Experimental water density is 1000 kg/m^3^, while the SPC/E model predicts 1008 kg/m^3^. Our simulation achieved an average density of 1021 ± 1 kg/m^3^, aligning well with both benchmarks and validating the protocol’s accuracy.

With thermal and pressure equilibrium established, positional restraints were released to initiate the 100-nanosecond MD simulation. This extensive timescale underscores the computational rigor of our approach. Long-range electrostatics were precisely handled via the Particle Mesh Ewald (PME) method with order 4, ensuring accurate treatment of electrostatic interactions. The software dynamically determines optimal processor allocation for PME calculations, balancing computational efficiency and resource utilization. GPU accelerators on modern supercomputers further enhanced performance, enabling unprecedented simulation speed while maintaining accuracy.

In essence, the combination of position restraint release, 100-ns MD simulation, optimized PME implementation, and GPU acceleration constitutes a robust computational pipeline. This framework ensures precise capture of system dynamics with exceptional accuracy and efficiency, validating the simulation’s scientific integrity.

### Unrestrained MD simulations

**FIGURE 6:**
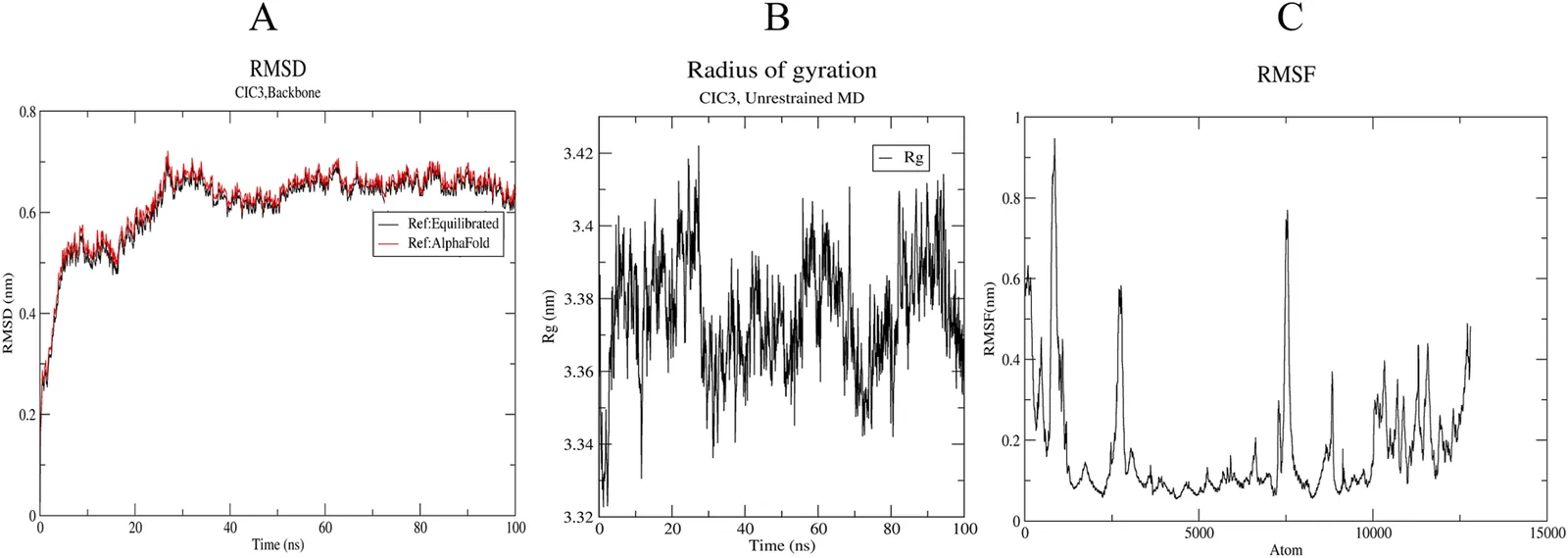
The RMSD, radius of gyration and RMSF values within 100ns MD simulation time. **(A)** The RMSD values extracted from the complex structure. **(B)** The radius of gyration values extracted from the complex structure. **(C)** The RMSF values extracted from the complex structure.

The slightly lower RMSD value for equilibrated Lig8 indicates that this ligand retains a stable configuration as illustrated in Figure 6A. This structural stability may lead to enhanced inhibitory efficacy against the ClC-3 protein.

Furthermore, our examination of ClC-3’s radius of gyration demonstrated a steady decline from 3.42 nm to 3.32 nm accompanied by a certain oscillation throughout the simulation timeline, as visualized in Figure 6B. This numerical range confirms the protein’s structural compactness.

To investigate ClC-3’s dynamic behavior more thoroughly, we computed the root mean square fluctuations (RMSF) of its atomic components using simulation trajectories. As illustrated in Figure 6, the equilibrated RMSD values, alongside the steady radius of gyration (Rg) and reduced RMSF profiles across the highly fluctuating regions, indicate that Lig8 retains a stable binding configuration and induces a more rigid, compact conformational state in the ClC-3 protein. Importantly, while these *in silico* metrics strongly support the structural stabilization of the ClC-3-Lig8 complex, they do not intrinsically conclude functional inhibitory efficacy. The computational stabilization observed here highlights Lig8 as a structurally viable binder; however, future *in vitro* functional assays, such as patch-clamp electrophysiology, are strictly required to confirm whether this stable binding translates into actual inhibitory action against the ClC-3 chloride channel.

To evaluate the dynamic binding behavior of the hit compound within the ClC-3 chloride ion channel, the initial binding pose predicted by GNINA docking (Figure 4A, purple spheres) and the fully relaxed conformation obtained after molecular dynamics (MD) simulations (Figure 4A, green spheres) were superimposed. A noticeable spatial deviation between the two poses was observed. This structural shift is primarily attributed to the intrinsic flexibility of the ClC-3 protein and the ‘induced fit’ conformational changes that occur in a dynamic, explicitly solvated environment. While initial molecular docking relies on a semi-rigid receptor to identify a preliminary binding site, the MD simulation allows the entire protein-ligand-solvent system to overcome kinetic barriers and relax into a more thermodynamically stable state. The transition from the docking pose to the final MD pose demonstrates that the ligand optimizes its position and orientation to maximize favorable intermolecular interactions (such as with residues GLY283, PHE527, and GLY584) within the dynamically evolving binding pocket.

#### MM/PBSA free energy calculation

**FIGURE 7:**
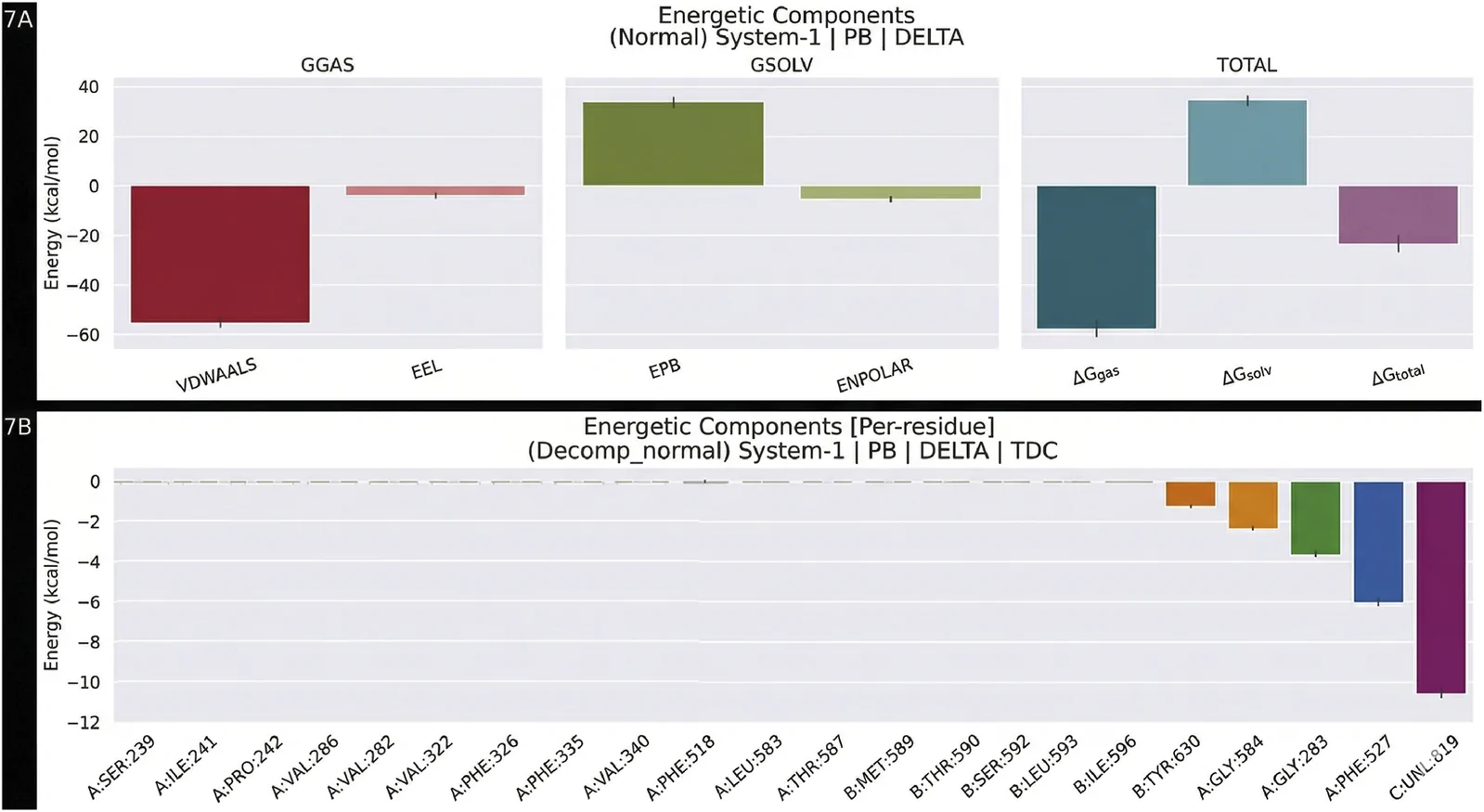
The energy decomposition details. **(A)** The illustration of energy difference for the receptor-ligand complex. **(B)** Per-residue decomposition energies for the ClC-3 protein using MM/PBSA. VDWAALS denotes the van der Waals energy, EEL denotes the electrostatic energy, ENPOLAR denotes the nonpolar solvation energy and EPB denotes the Poisson-Boltzmann energy.

Using the MM/PBSA methodology, we quantitatively evaluated the thermodynamic stability of the ClC-3 and Lig8 complex. This approach, executed via the gmx_MMPBSA program, provides a rigorous perspective on the driving forces behind the protein-ligand interactions. As detailed in Table 5 and visually confirmed in Figure 7A, the total binding free energy (ΔG_total_) of the complex reached a highly favorable −23.51 kcal/mol, indicating a spontaneous and stable binding process. The energy component analysis reveals that intermolecular van der Waals interactions act as the primary driving force for complex formation. While the polar solvation free energy introduces an unfavorable penalty to the binding, it is substantially overcome by the highly favorable gas-phase energy (ΔG_gas_ = −58.34 kcal/mol) and the nonpolar solvation term.

**TABLE 5:** Summary of binding free energy calculated by MM/PBSA (kcal/mol).

Energy component	Average	σ	SEM
ΔG_gas_	−58.34	1.76	1.21
ΔG_solv_	34.83	1.06	0.56
ΔG_total_	−23.51	0.93	1.03

To gain deeper insights into the specific contributions of individual amino acids to the overall binding affinity, a per-residue free energy decomposition was performed (Figure 7B). The energetic landscape perfectly aligns with our prior structural observations. Consistent with the docking analysis, residue PHE527 exhibits the most prominent favorable energy contribution among the protein residues, primarily dominated by van der Waals forces. Furthermore, residues GLY283 and GLY584 also provide significant energetic stabilization. These crucial residues actively participate in forming a robust binding interface that tightly anchors Lig8, thereby restricting the channel’s conformational dynamics and underpinning the compound’s inhibitory mechanism against ClC-3.

## Discussion

In this study, we utilized an integrated computational approach to identify novel small-molecule modulators of the ClC-3 chloride channel. ClC-3 acts as a crucial anion gatekeeper, enabling chloride efflux during cellular stress, such as cellular swelling. This role is critical for maintaining cellular homeostasis against osmotic disturbances and ensuring optimal cellular function. Through our virtual screening pipeline, we examined a massive repository exceeding 180,000 compounds. Following stringent validation, including deep-learning rescoring and ADMET profiling, ZINC000001556308 (Lig8) was selected as the top candidate. Detailed atomistic analyses confirmed its robust binding conformation and stable molecular interactions with the ClC-3 channel.

The integration of traditional docking with 3D-CNN deep learning rescoring robustly minimized false positives in our pipeline. The performance of ZINC000001556308(Lig8), which closely mirrored the established positive control IAA-94 in GNINA evaluations, strongly underscores its potential as a novel scaffold for ClC-3 targeted therapy.

To elucidate the mechanism of ZINC000001556308's interaction with ClC-3, molecular dynamics (MD) simulations were deployed. These simulations provided insights into key parameters including root-mean-square deviation (RMSD), radius of gyration, and root-mean-square fluctuation (RMSF). Comprehensive evaluations demonstrated ZINC000001556308's enhanced inhibitory capabilities.

This pivotal discovery positions ZINC000001556308 as a promising computational lead for cervical cancer. Its ability to precisely target ClC-3 establishes it as a standout contender in pharmaceutical innovation. This research not only advances understanding of ZINC000001556308's inhibitory pathways but also pioneers new therapeutic strategies for cervical cancer and potentially related diseases.

The discovery of Lig8 holds substantial clinical significance, particularly in the context of overcoming multidrug resistance in cervical cancer. Clinically, cisplatin-based chemotherapy remains the standard of care; however, acquired resistance often leads to treatment failure and relapse. Because overexpressed ClC-3 drives cisplatin resistance through lysosomal alkalinization and the dysregulation of autophagy, selectively inhibiting ClC-3 with Lig8 could restore normal intracellular pH gradients and re-sensitize resistant cancer cells to conventional chemotherapeutics. Therefore, Lig8 could be explored clinically not merely as a monotherapy, but as a potent chemosensitizer in combination regimens. Furthermore, the favorable ADMET profile and absence of hERG toxicity, mutagenicity, or carcinogenicity associated with Lig8 suggest a wide therapeutic window, potentially minimizing the severe off-target side effects typical of systemic chemotherapy. Looking forward, these *in silico* findings lay a robust foundation for subsequent translational research, including the validation of Lig8’s efficacy and safety in patient-derived organoids (PDOs) and *in vivo* tumor xenograft models.

In conclusion, driven by the critical motivation to overcome the lack of targeted therapies for ClC-3-mediated oncogenic signaling, this study successfully establishes a highly efficient, AI-enhanced computational framework. By shifting our methodological approach to an AIDD pipeline, we harnessed the advantages of 3D-CNN rescoring to efficiently filter the ZINC15 library, overcoming significant computational hardware constraints while maintaining strict evaluation criteria. This proposed scheme led to the discovery of Lig8 and the identification of a previously uncharacterized binding pocket anchored by key residues GLY283, PHE527, and GLY584. These findings not only highlight the novelty and academic merit of integrating deep learning with molecular dynamics in ion channel drug discovery, but also provide a structurally validated chemical scaffold for future translational therapeutics against cervical cancer.

### Limitations of the study

While this study provides a robust computational framework for identifying ClC-3 modulators, several limitations must be acknowledged. First, all findings are derived from *in silico* screening and remain strictly hypothesis-generating until validated by *in vitro* functional assays, such as patch-clamp electrophysiology or halide efflux assays, which are beyond the computational scope of this current work. Second, our docking protocol utilized an AlphaFold-predicted structure; future structural validations should benchmark these poses against recently resolved high-resolution cryo-EM structures (e.g., PDB ID 9DO0). Third, due to computational resource constraints, an apo ClC-3 MD simulation was not conducted as a baseline control, and screening against other chloride channel families was not performed, leaving potential off-target effects uncharacterized. Finally, the inherent limitations of computational methods—including docking scoring inaccuracies, the finite 100-ns timescale of molecular dynamics, and the probabilistic nature of ADMET predictions—should be considered when interpreting the translational impact of ZINC000001556308 (Lig8).

## Data Availability

The original structures, chemical libraries, and predicted structural models analyzed during the current study are available in the Uniprot, AlphaFold, and ZINC15 databases. All critical computational configurations have been detailed in the Materials and Methods section to ensure reproducibility.
